# Supplementary material for: The metronomic combination of paclitaxel with cholinergic agonists inhibits triple negative breast tumor progression. Participation of M2 receptor subtype
Source: PLoS One. 2020 Sep 10;15(9):e0226450. doi: 10.1371/journal.pone.0226450 (PMC7482849; doi:10.1371/journal.pone.0226450)

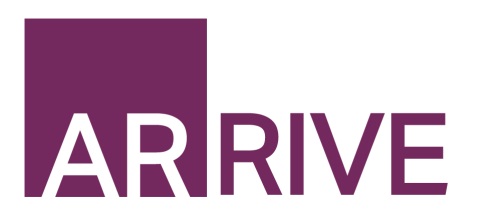


The ARRIVE Guidelines Checklist

Animal Research: Reporting In Vivo Experiments

Carol Kilkenny^1^, William J Browne^2^, Innes C Cuthill^3^, Michael Emerson^4^ and Douglas G Altman^5^

*^1^The National Centre for the Replacement, Refinement and Reduction of Animals in Research, London, UK, ^2^School of Veterinary Science, University of Bristol, Bristol, UK, ^3^School of Biological Sciences, University of Bristol, Bristol, UK, ^4^National Heart and Lung Institute, Imperial College London, UK, ^5^Centre for Statistics in Medicine, University of Oxford, Oxford, UK.*

|  | | ITEM | RECOMMENDATION | Section/ Paragraph |
| --- | --- | --- | --- | --- |
| 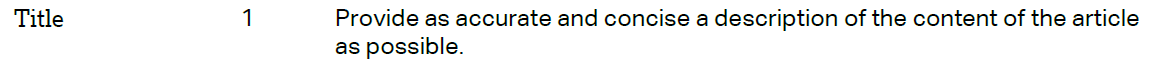 | | | Title. Page 1. |  |
| 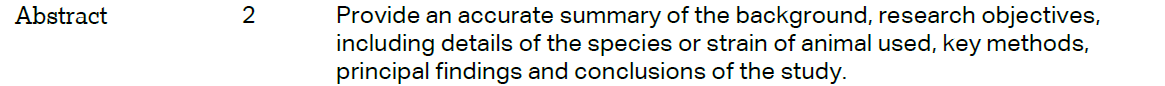 | | | Abstract. Page 2. |  |
| INTRODUCTION | | |  |  |
| 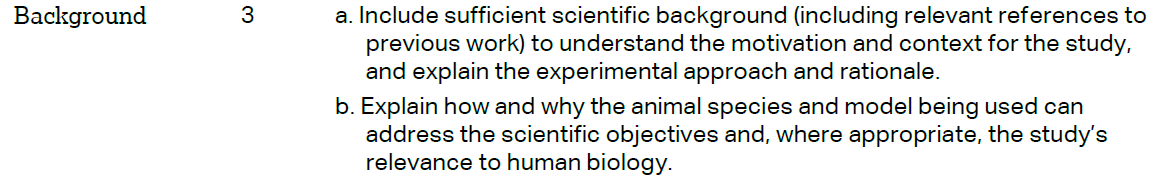 | | | a. Introduction, paragraph 1,2.  b.Materials and methods, paragraph 7 (Tumor-induced angiogenesis) |  |
| 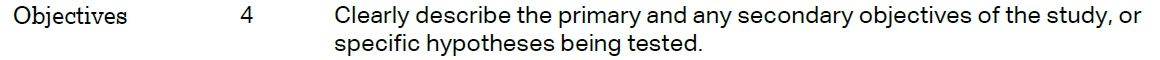 | | | Introduction, paragraph 3. |  |
| METHODS | | |  |  |
| 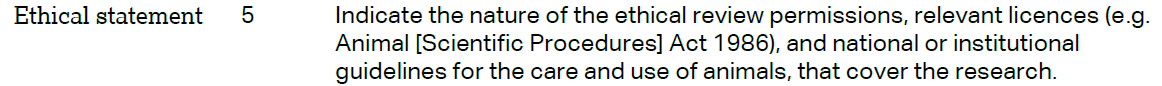 | | | Materials and methods, paragraph 7. (Tumor-induced angiogenesis) |  |
| 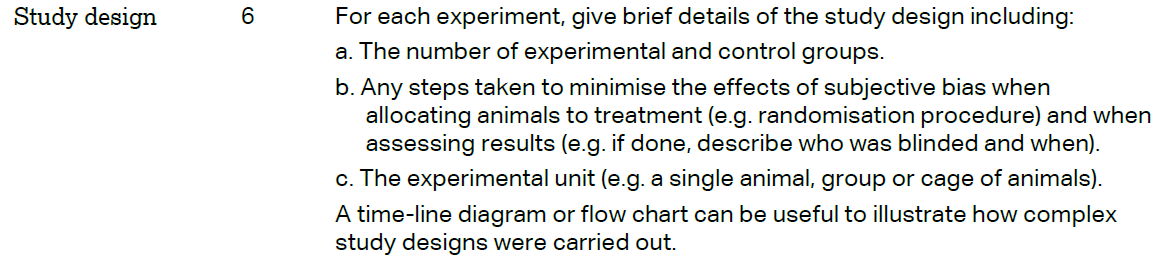 | | | a, b. Materials and methods, paragraph 8.  c, Materials and methods, paragraph 9.  The study design is not as complex to present a flux chart. |  |
| 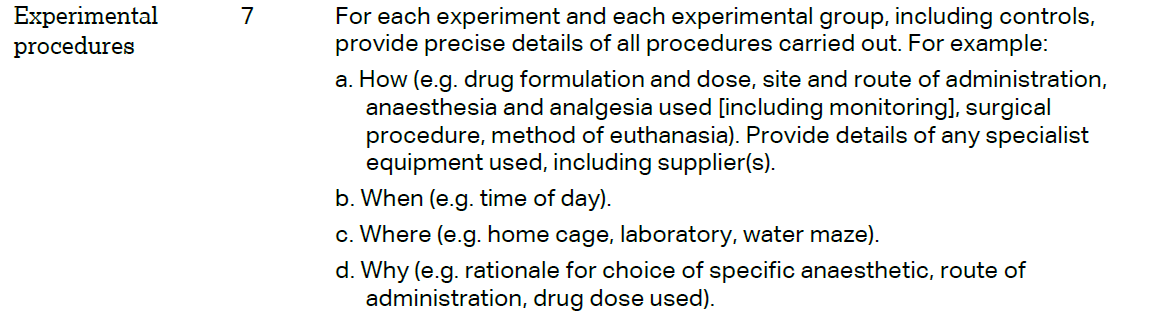 | | | a. Materials and methods, paragraph 8.  b. The time of the injections is not relevant for this study.  c. Materials and methods, paragraph 8.  d. We do not use anaesthetic because the animals do not suffer considering the small volumes inoculated. |  |
| 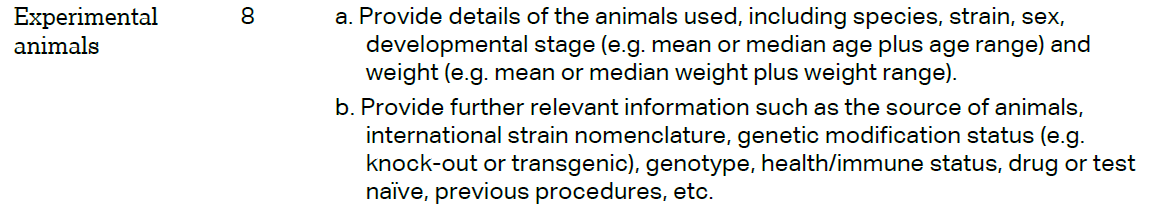 | | | a, b. Materials and methods, paragraph 7. |  |

The ARRIVE guidelines. Originally published in *PLoS Biology*, June 2010^1^

| 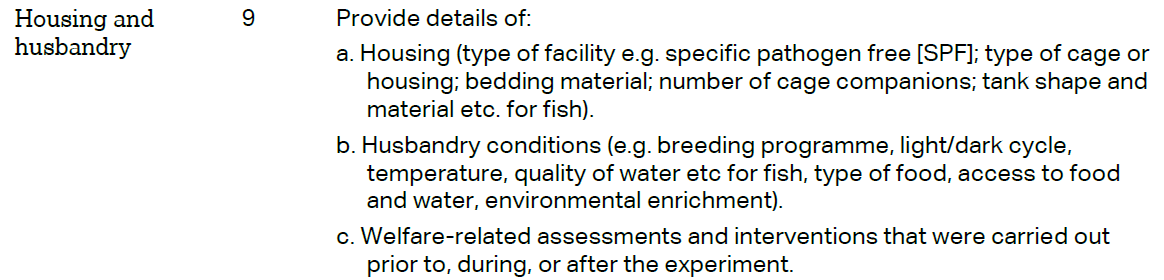 | a,b,c. Materials and methods, paragraph 7. | |
| --- | --- | --- |
| 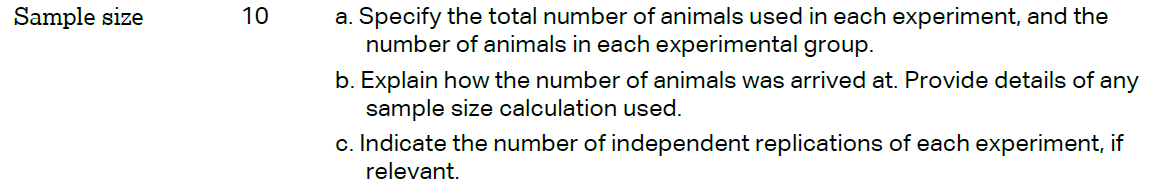 | a, c. Materials and methods, paragraph 8.  b. It is the minimal number of animals that must be used to obtain significant differences | |
| 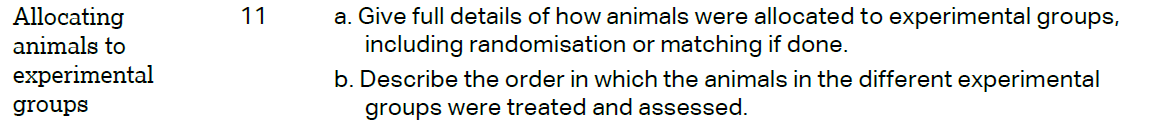 | a. Materials and methods, paragraph 8.  b. The order in which the animals were treated is not relevant | |
| 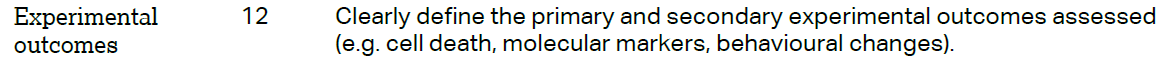 | Materials and methods, paragraph 9. | |
| 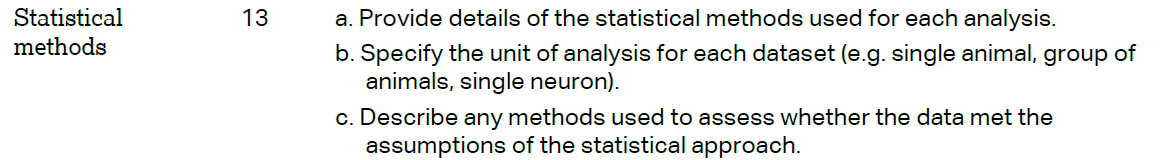 | a, c. Materials and methods, paragraph 11.  b. Materials and methods, paragraph 9. | |
| RESULTS |  | |
| 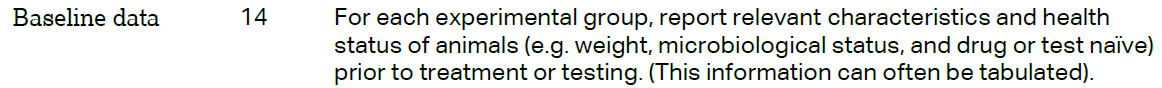 | Materials and methods, paragraph 7. | |
| 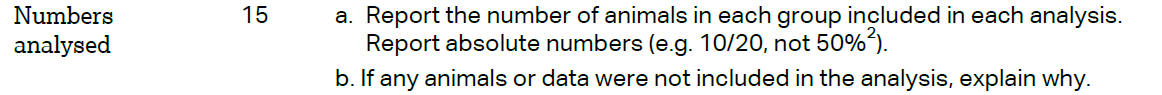 | a. Materials and methods, paragraph 8.  b. All of the animals were included in the analysis. | |
| 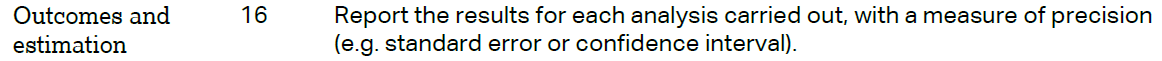 | Results, paragraph 11. | |
| 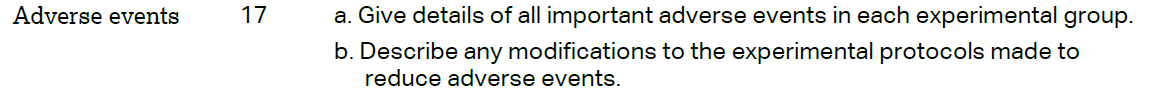 | a. No important adverse events occurred.  b. No modification to the protocols was made | |
| DISCUSSION |  | |
| 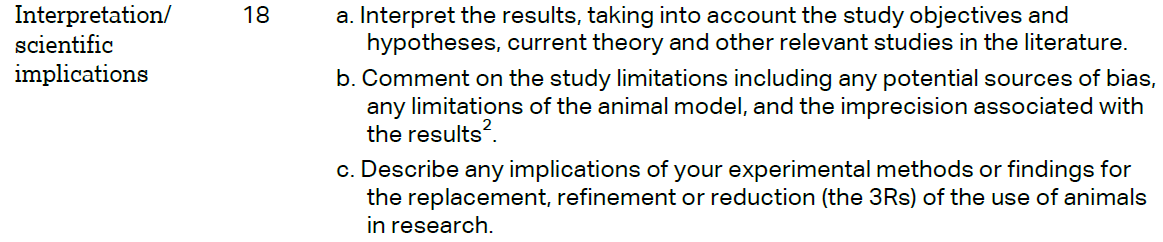 | a, b, Discussion and conclusions, paragraph 7.  c. The 3Rs rule is already applied in the experimental designed. | |
| 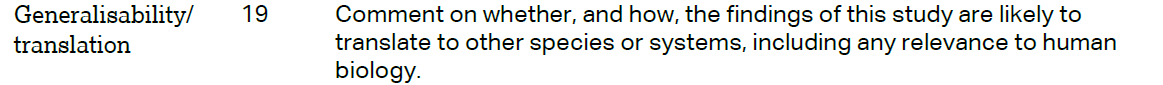 | a. Discussion and conclusions, paragraph 6, 7. | |
| 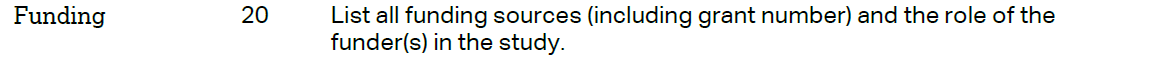 | | CONICET PIP 201501 00239; ANPCYT PICT 2396; UBA UBACYT 20020130100168BA |
| oni | |  |


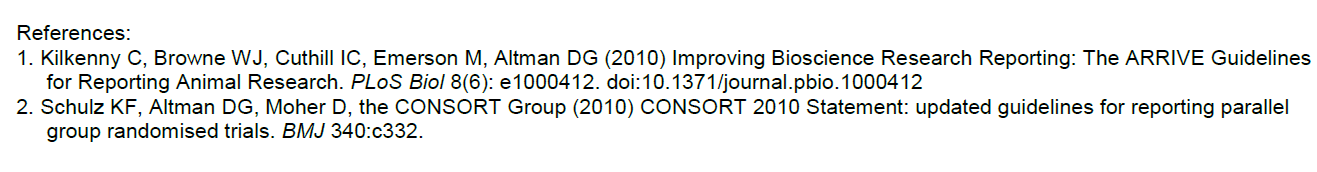

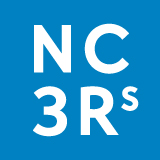

Supplement: S1 Checklist — (DOCX) [file pone.0226450.s001.docx]
