# Supplementary material for: The metronomic combination of paclitaxel with cholinergic agonists inhibits triple negative breast tumor progression. Participation of M2 receptor subtype
Source: PLoS One. 2020 Sep 10;15(9):e0226450. doi: 10.1371/journal.pone.0226450 (PMC7482849; doi:10.1371/journal.pone.0226450)
Supplement: S1 Raw images — (PDF) [file pone.0226450.s003.pdf]

FIG 1

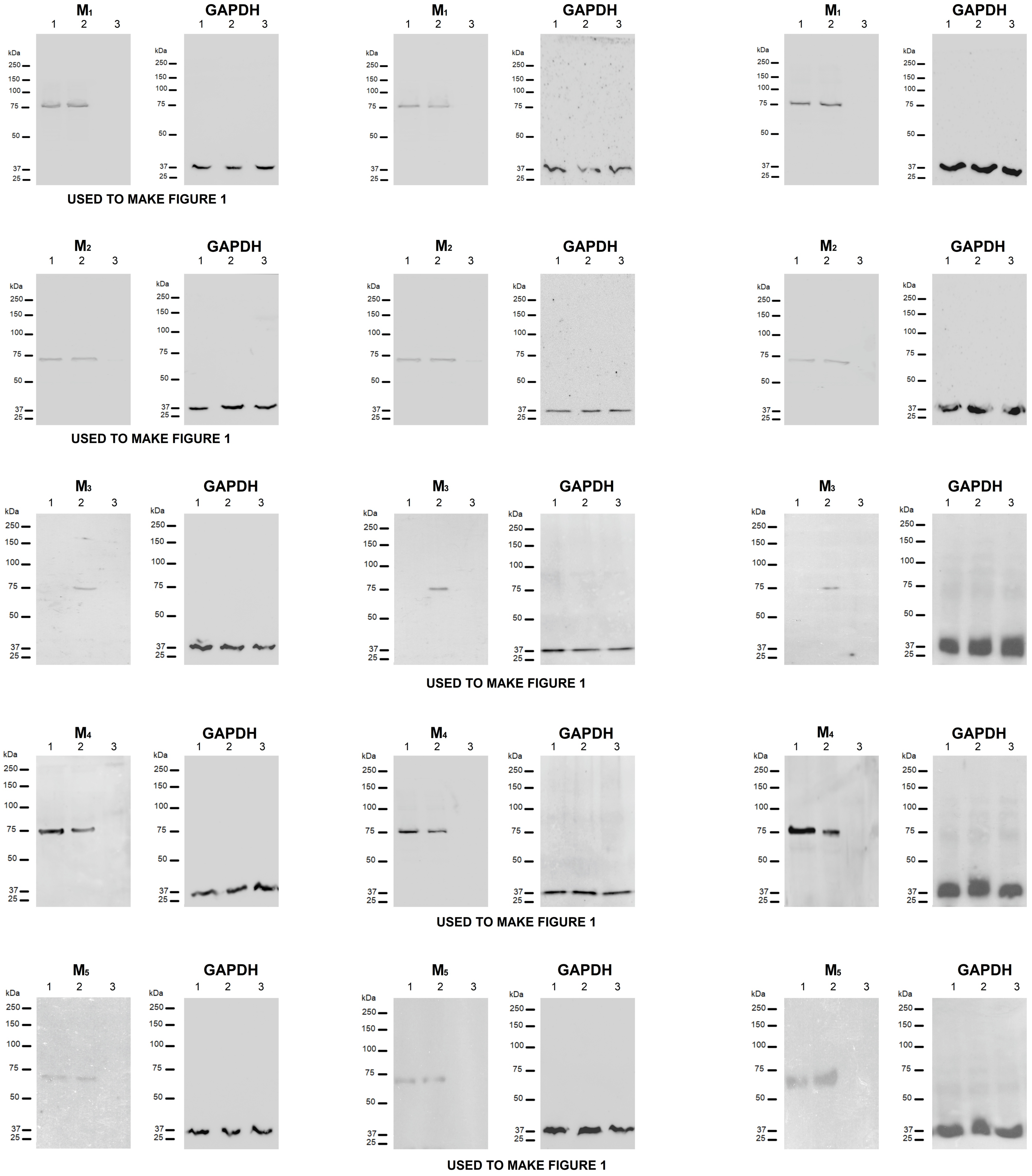

1: MDA-MB 231 cells
2: MDA-MB468 cells
3: MCF-10A cells

FIG 6A AND 6C

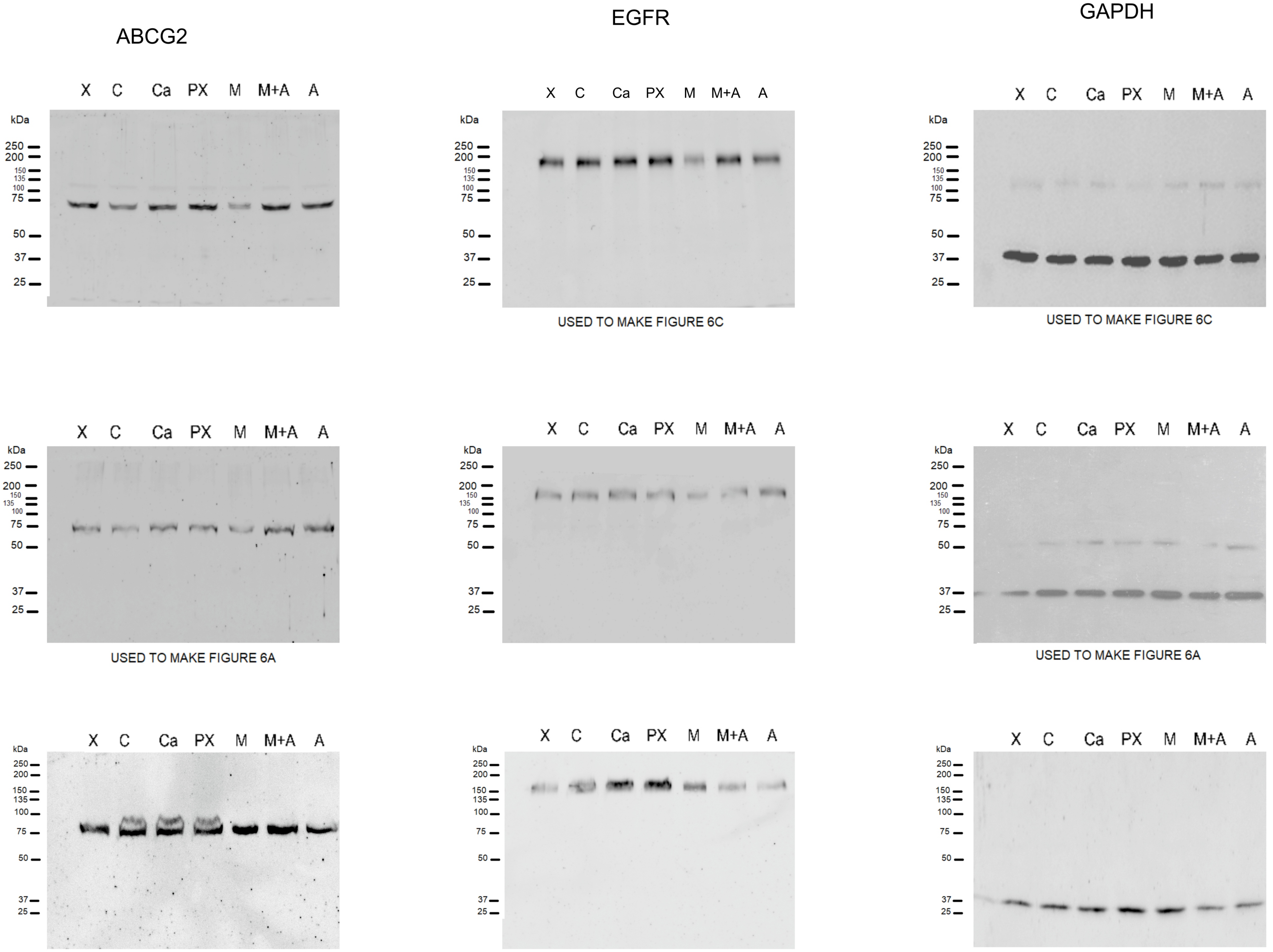

X: LANE NOT INCLUDED
C: CONTROL
Ca: CARBACHOL (8.6x10^{-12}M)
PX: PACLITAXEL (10^{-8} M)
M: Ca+PX
M+A: M+ATROPINE (10^{-9} M)
A: ATROPINE (10^{-9} M)

FIG 6B AND 6D

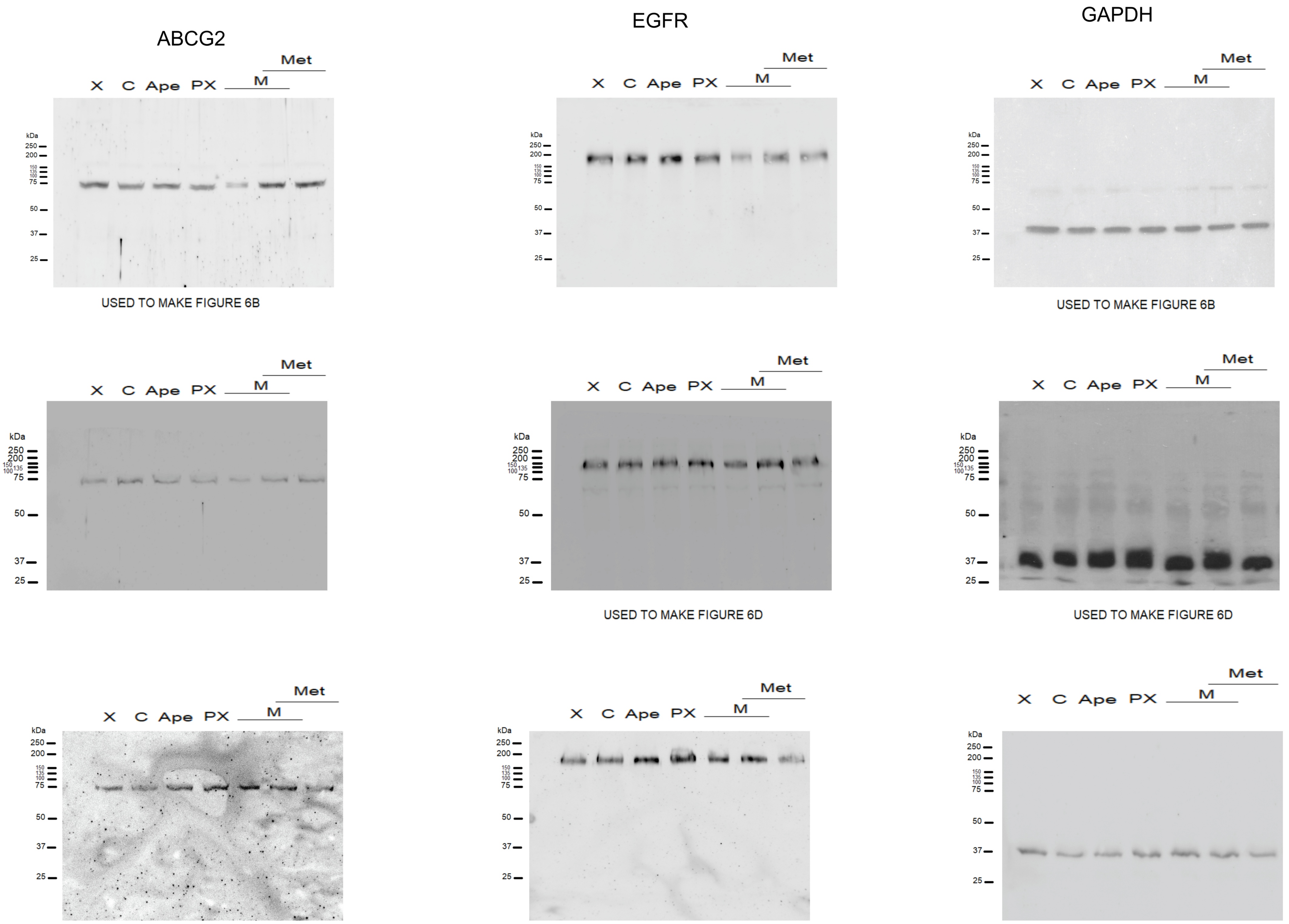

X: LANE NOT INCLUDED
C: CONTROL
Ape: ARECAIDINE PROPARGYL ESTER (1,1X10^{-5} M)
PX: PACLITAXEL (10^{-8} M)
M: Ape+PX
M+Met: M+METHOCTRAMINE (10^{-5} M)
Met: METHOCTRAMINE (10^{-5} M)

FIG 8A

VEGF-A

GAPDH

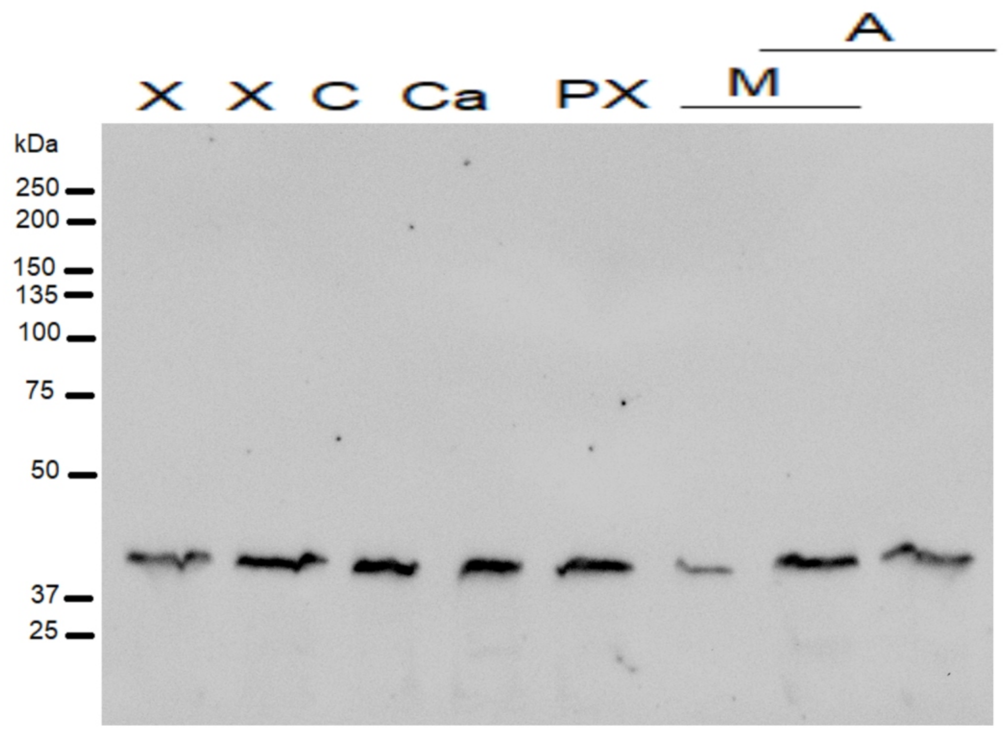

USED TO MAKE FIGURE 8A

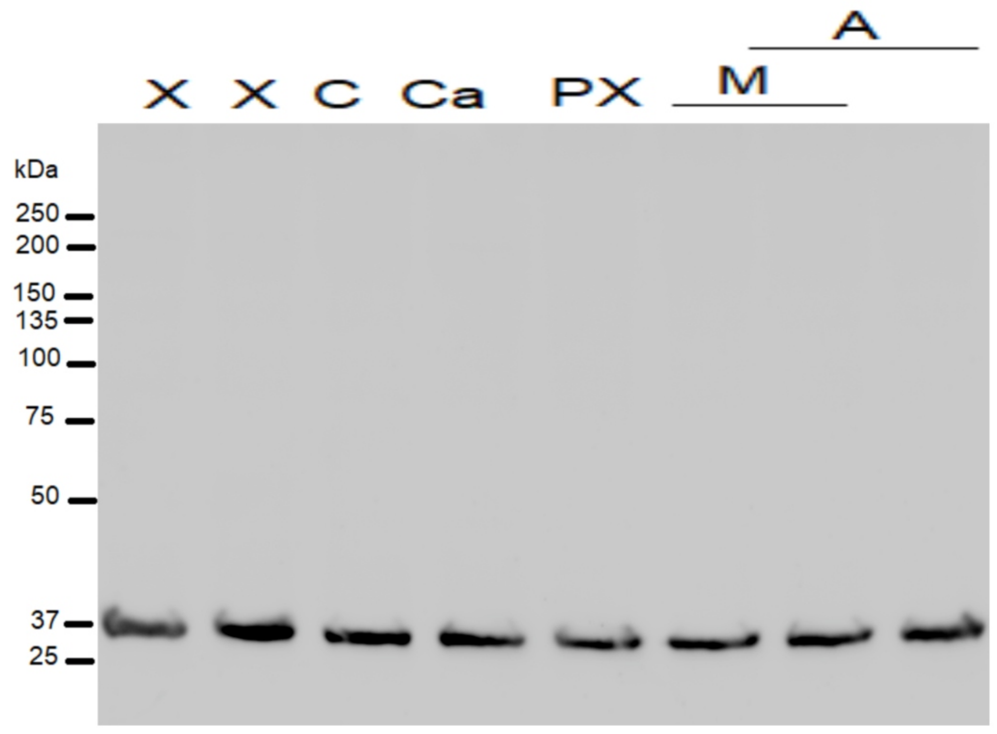

USED TO MAKE FIGURE 8A

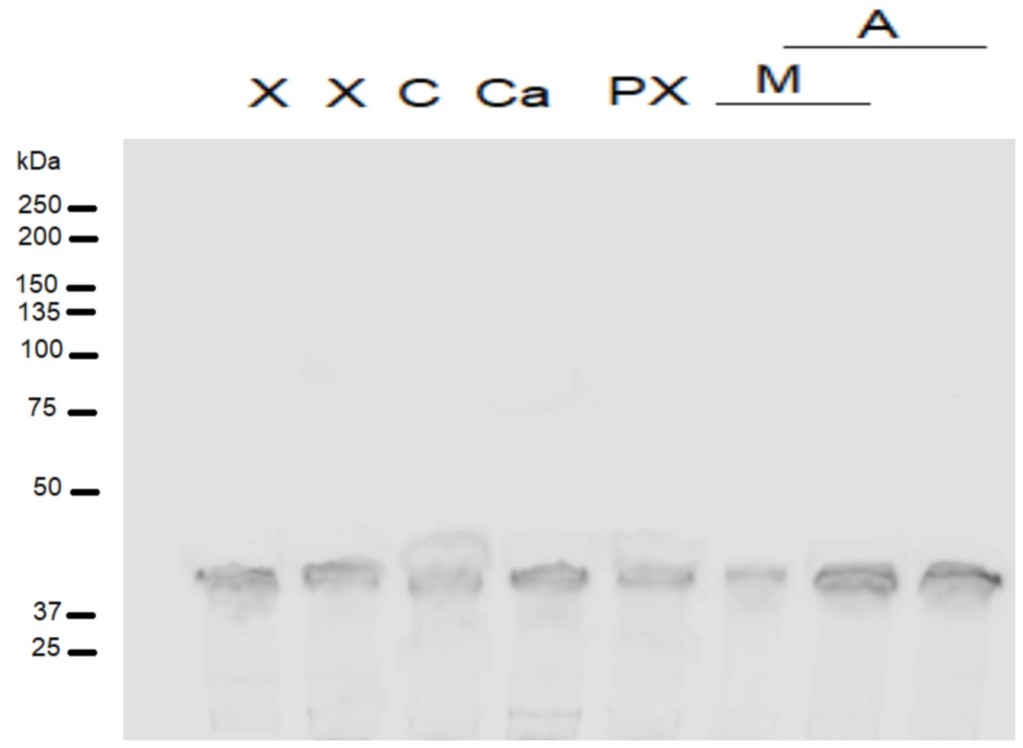

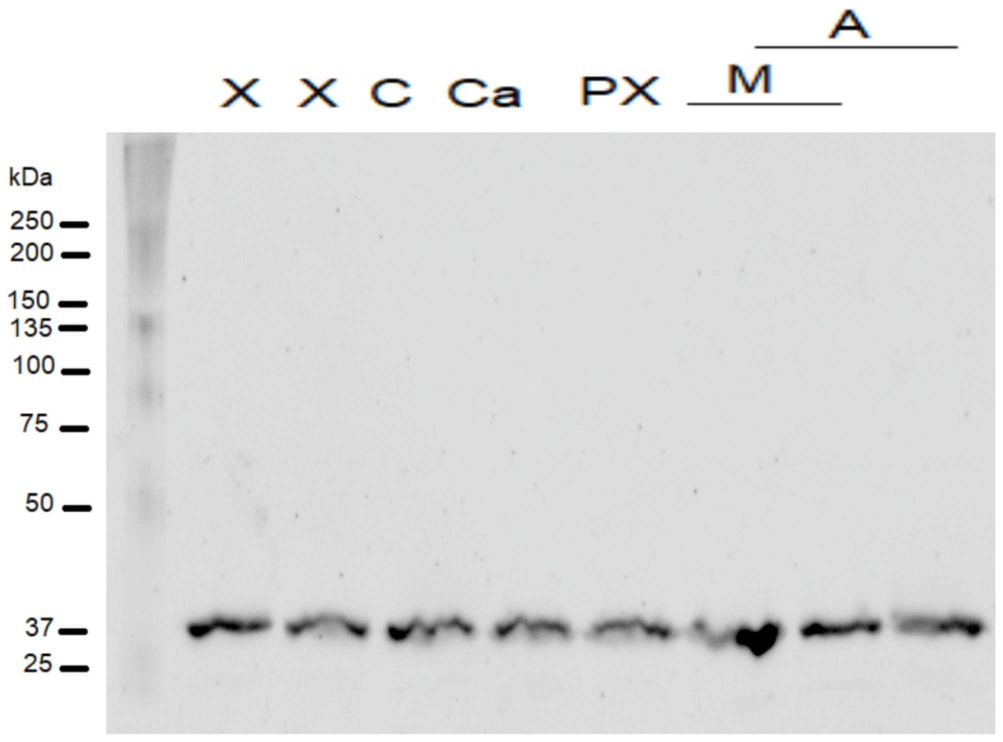

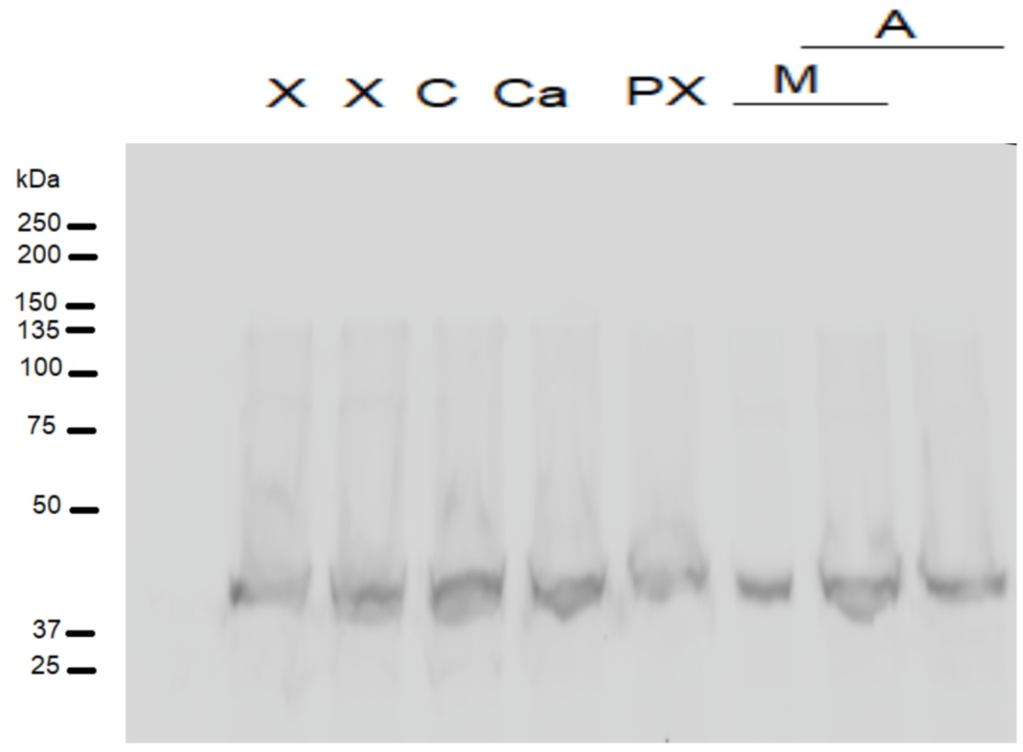

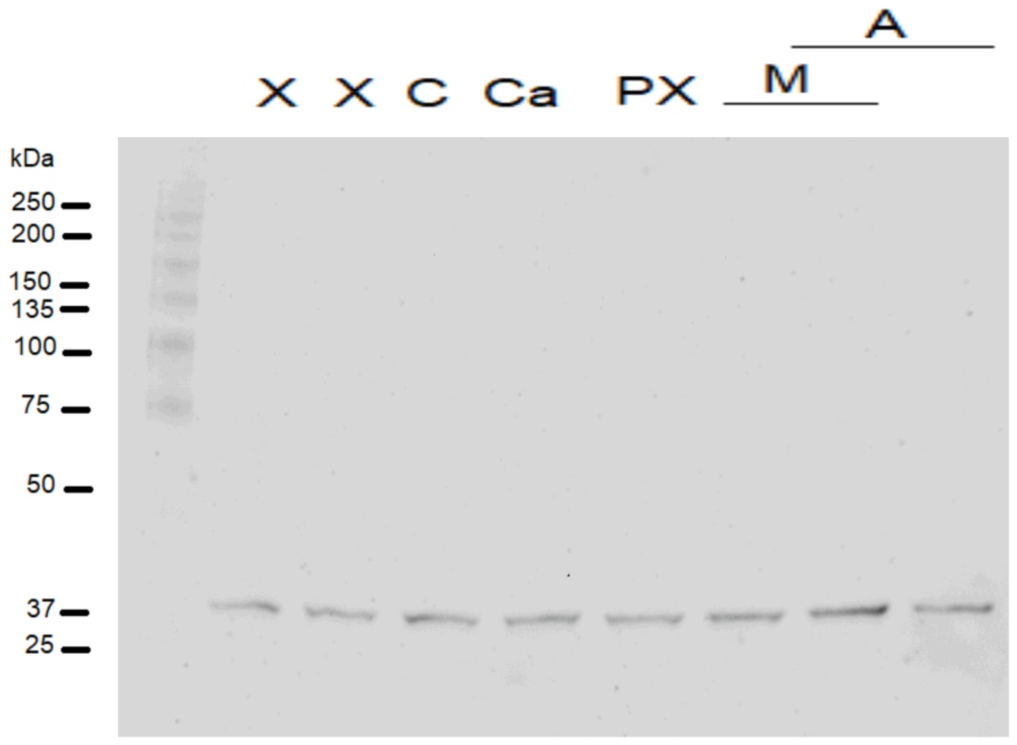

X: LANE NOT INCLUDED
C: CONTROL
Ca: CARBACHOL (8.6X10^{-12} M)
PX: PACLITAXEL (10^{-8} M)
M: Ca+PX
M+A: M+ATROPINE (10^{-9} M)
A: ATROPINE (10^{-9} M)

FIG 8B

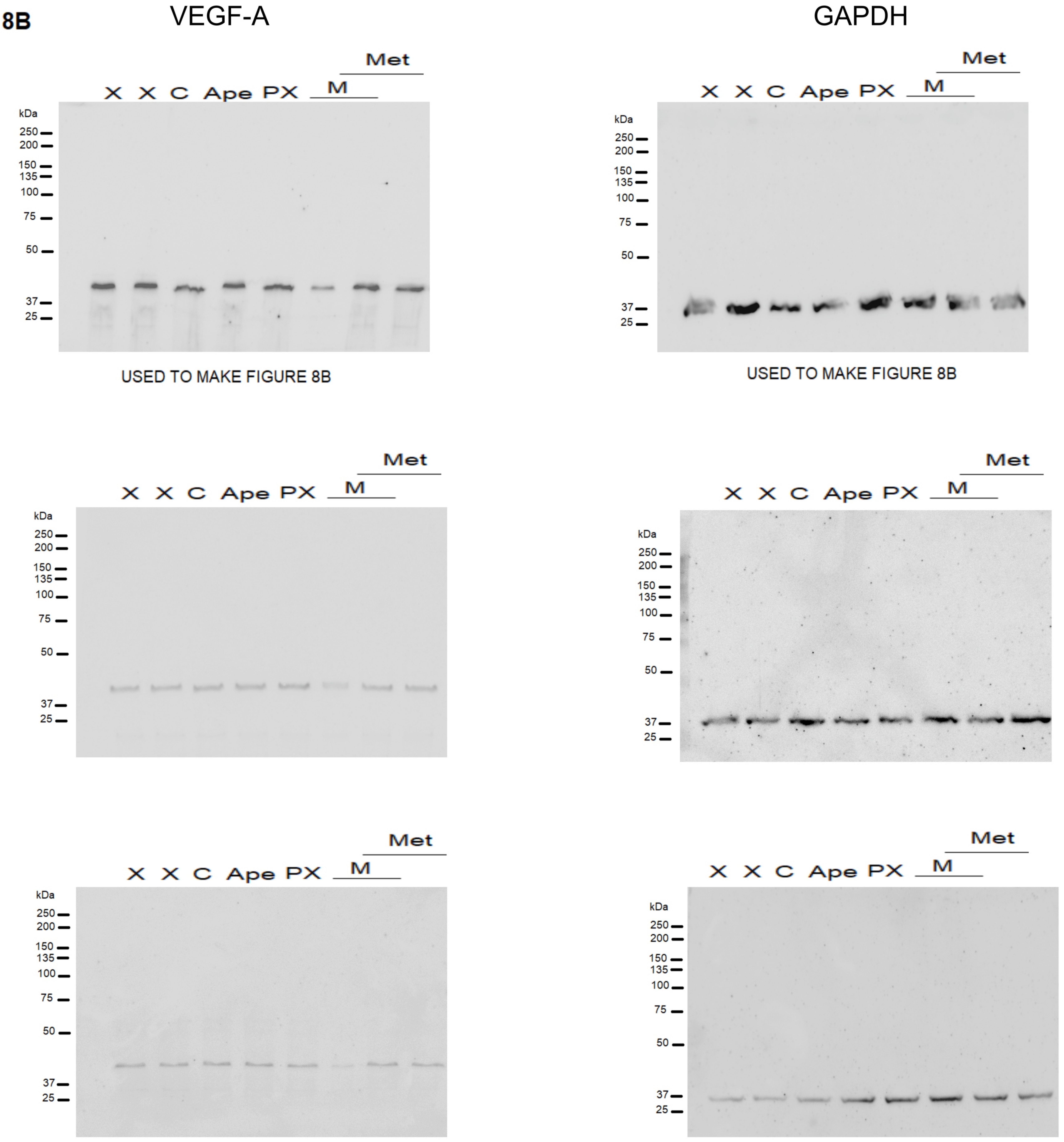

X: LANE NOT INCLUDED
C: CONTROL
Ape: ARECAIDINE PROPARGYL ESTER ($1.1X10^{-5}$ M)
PX: PACLITAXEL (10^{-8} M)
M: Ape+PX
M+Met: M+METHOCTRAMINE (10^{-5} M)
Met: METHOCTRAMINE (10^{-5} M)
