# Supplementary figures and images for: The metronomic combination of paclitaxel with cholinergic agonists inhibits triple negative breast tumor progression. Participation of M2 receptor subtype
Source: PLoS One. 2020 Sep 10;15(9):e0226450. doi: 10.1371/journal.pone.0226450 (PMC7482849; doi:10.1371/journal.pone.0226450)

S1 Fig

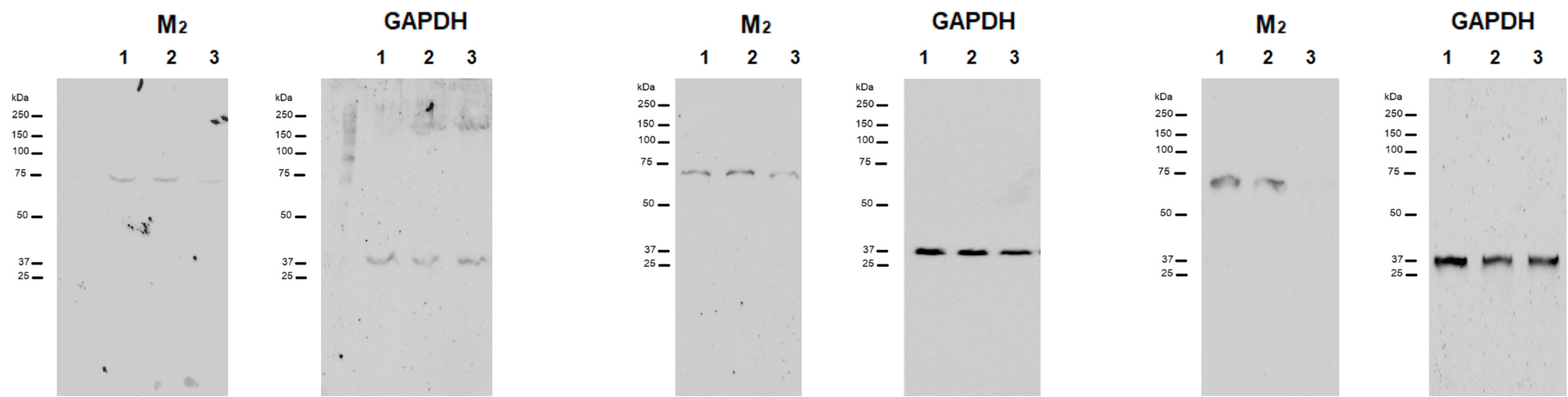

USED TO MAKE SUPPLEMENTARY FIGURE 1

1: MDA-MB231 cells  
2: MDA-MB231 cells + NC-siRNA  
3: MDA-MB231 cells + M2-siRNA

Supplement: S2 Raw images — (PDF) [file pone.0226450.s004.pdf]
