# Supplementary material for: The metronomic combination of paclitaxel with cholinergic agonists inhibits triple negative breast tumor progression. Participation of M2 receptor subtype
Source: PLoS One. 2020 Sep 10;15(9):e0226450. doi: 10.1371/journal.pone.0226450 (PMC7482849; doi:10.1371/journal.pone.0226450)
Supplement: Attachment — Submitted filename: Response to Reviewers.docx [file pone.0226450.s007.docx]

**We thank the editor and reviewers for your useful suggestions that helped our manuscript improve.**

**Response to academic editor and reviewers**

**1. We noticed you have some minor occurrences of overlapping text…**

We rephrased or cited all duplicated text found in the manuscript.

We rephrased the following sections (line number corresponds to “Manuscript with track Changes”):

**In Material and Methods; Cell culture:**

- We replaced “obtained” by “acquired”. Line 105.
- We replaced “purchased” by “bought”. Line 109.
- We replaced “detached” by “removed”. Line 113.
- We replaced “replaced” by “changed”. Line 115.
- We replaced “assayed” by “determined”. Line 116.
- We replaced “confirmed” by “observed”. Line 117.

**In Material and Methods; Detection of muscarinic receptors by Western blot:**

- We replaced paragraph from line 120 to line 135 by paragraph from line 136 to line 151.

**In Material and Methods; Cell viability assay:**

- We replaced paragraph from line 155 to line 175 by paragraph from line 176 to line 196.

**In Material and Methods; Tumor-induced angiogenesis:**

- We replaced paragraph from line 236 to line 255 by paragraph from line 256 to line 276.

The following references were added:

- Sales ME, Español A, Salem A, Pulido P, Sanchez Y, Sanchez F. Role of muscarinic acetylcholine receptors in breast cancer. Design of metronomic chemotherapy. Curr Clin Pharmacol. 2019; 14: 91-100.
- Schettini F, Buono G, Cardelasi C, Desideri I, de Palacios S, Del Mastro L. Hormone receptor/human epidermal growth factor receptor 2-positive breast cancer: where we are now and where we are going. Cancer Treat Rev. 2016; 46: 20-26.
- Sales ME. Muscarinic receptors as targets for metronomic therapy in breast cancer. Curr Pharm Des. 2016; 22: 2170-2177.
- Dai X, Zhang J, Guo G, Cai Y, Cui R, Yin C, et al. A mono-carbonyl analog of curcumin induces apoptosis in drug-resistant and mitochondrial dysfunction. Cancer Manag Res. 2018; 10: 3069-3082.
- Hu T, Li Z, Gao CY, Cho CH. Mechanisms of drug resistance in colon cancer and its therapeutic strategies. World J Gastroenterol. 2016; 22: 6876-6889.

The following reference was removed:

- Hayes DF, Thor AD, Dressler LG, Weaver D, Edgerton S, Cowan D, et al. [Cancer and Leukemia Group B (CALGB) Investigators](https://www.ncbi.nlm.nih.gov/pubmed/?term=Cancer%20and%20Leukemia%20Group%20B%20(CALGB)%20Investigators%5BCorporate%20Author%5D). HER2 and response to paclitaxel in node-positive breast cancer. N Engl J Med. 2007; 357:1496-506.

**2. As part of your revision, please complete and submit a copy of the ARRIVE Guidelines checklist.**

We attached the corresponding file named “ARRIVE Guidelines PONE-D-19-32678”.

**3. Upon re-submitting your revised manuscript, please upload your study´s minimal underlying data set.**

We attached the corresponding excel file named “S2 minimal data set”.

**4. PLOS ONE now requires that authors provide the original uncropped and unadjusted images underlying all blot or gel results reported.**

We attached the corresponding PDF file named “S1 raw images”.

**5. Review Comments to the Author**

Please use the space provided to explain your answers to the questions above. You may also include additional comments for the author, including concerns about dual publication, research ethics, or publication ethics. (Please upload your review as an attachment if it exceeds 20,000 characters)

**Reviewer #1:** In this study authors demonstrate the role of metronomic therapy employing a traditional anti-cancer drug, paclitaxel plus muscarinic agonists at low doses on Triple negative tumors. In general, this study is well-performed and qualified to accept to PLOS ONE for publication if the authors address two major comments as below:

**1. It is recommended to run all the samples of proteins in the single gel in Figure-1A and Figure-1B and present the western blot data together and not individually.**

Western blot assays for muscarinic receptors/GAPDH were done again and we made the Figure according reviewer´s suggestions (Fig 1).

2**. GAPDH levels for MCF10A cells are not comparable with MDAMB231 cells. It seems that the amount of total protein is less in the MCF10 cells thus the comparison M-receptors are not accurate. This experiment needs to be done more convincingly.**

See response in item 1, please.

**Reviewer #2:** The manuscript entitled “The metronomic combination of paclitaxel with cholinergic agonists inhibits triple-negative breast tumor progression. Participation of M2 receptor subtype”. Espanol AJ and colleagues were trying to identify the role of muscarinic receptors on cancer cell growth and migration. In this study, authors used carbachol or arecaidine propargyl ester along with traditional anti-cancer drugs paclitaxel and their inhibitory effect on breast cancer cells. Also, they have shown the chemoresistance mechanism due to the overexpression of ABCG2 and EGFR expression.

**1. EGFR activation is more important than just expression, moreover, EGFR signaling is highly activated in other breast cancer subtypes rather than TNBC.**

Even though that the activation of EGFR is important in the regulation of tumor growth; several authors consider that the up-regulation of EGFR expression is a bad prognosis marker and an indicator of chemoresistance in triple negative breast cancer (TNBC) patients or cell lines:

a. Zhang et al., Biomed Res Int. 2015;2015:357485 pointed to the distinct expression of EGFR and the prevalence of BRCA1 mutation indicating that EGFR, and BRCA1 might be unique biomarkers for targeted therapy and prognosis in TNBC.
b. Turner et al., Sci Rep. 2020 Jan 30;10(1):1493 concluded that EGFR is highly expressed in basal-like PDXs, cell lines, and patients, and high expression of this gene reduces metastasis-free survival, suggesting that targeting of this protein holds promise for potential clinical success in TNBC.
c. Islam et al., in J Cell Physiol. 2020 Jan 20. doi: 10.1002/jcp.29466 indicate that, the chemotolerance mechanism of TNBC has not yet been studied in detail. Infrequent messenger RNA expression, gene amplification (10-32.5%), and mutation (1%) of EGFR were seen in the TNBC samples irrespective of therapy, suggesting the importance of EGFR protein stabilization in this tumor. Thus, this study showed that reduced nuclear expression of Y654-p-β-catenin in NACT samples due to down-regulation of EGFR protein through promoter hypomethylation-mediated up-regulation of SH3GL2, resulting in low proliferation index/CD44 prevalence with better prognosis of the NACT patients, might have an important role in the chemotolerance of TNBC.
d. Fleisher et al., Breast Cancer (Dove Med Press). 2019 Jul 23; 11:231-241 postulated that EGFR is frequently overexpressed in TNBC, and the EGFR-overexpressing TNBC presumably escapes EGFR inhibitor therapy by up-regulating autophagy and inhibiting apoptosis. To parse the autophagy-apoptosis crosstalk pathway as a potential targeted therapy in TNBC, the activity of an EGFR inhibitor, osimertinib, alone and in combination with an autophagy inhibitor, chloroquine, was examined in EGFR-overexpressing TNBC cell line, MDA-MB-231.

**2. Authors need to provide full blot rather than crop in Figure 1 and sup Fig.1.**

It is not possible to make Western blot assays using antibodies against the 5 muscarinic receptor subtypes in the same individual membrane, at the same time, because they have very similar molecular weights which makes difficult their identification.

See response in item 1 to Reviewer 1, please.

**3. Bar diagram would be a better representation of the quantification of wound healing experiments (Figure 7A and B).**

We changed Figure 7A-B by bar graphics according reviewer´s suggestions.

**4. In Figure 8A and B, the expression of VEGF is not showing any clear difference.**

We made new Western blot assays according reviewer´s suggestions. The Images included in Fig 8A-B shows the differences among experimental groups.
